# Evaluation of the retinal nerve fiber layer and ganglion cell complex thicknesses in patients with exfoliation syndrome

**DOI:** 10.3906/sag-1803-27

**Published:** 2019-02-11

**Authors:** Cem ALAY, Oya TEKELİ, Özge YANIK ODABAŞ, Feyza ÇALIŞ KARANFİL

**Affiliations:** 1 Tavşanlı Doç. Dr. Mustafa Kalemli State Hospital, Kütahya Turkey; 2 Department of Ophthalmology , Faculty of Medicine, Ankara University, Ankara Turkey; 3 Alaaddin Keykubat University Alanya Education and Research Hospital, Antalya Turkey; 4 Yüksek İhtisas University Koru Hospital, Ankara Turkey

**Keywords:** Exfoliation syndrome, retinal ganglion cell complex, retinal nerve fiber layer, optical coherence tomography

## Abstract

**Background/aim:**

This study aimed to evaluate retinal nerve fiber layer (RNFL) and ganglion cell complex (GCC) thicknesses using spectral domain optical coherence tomography (SD-OCT) in both unilateral and bilateral exfoliation syndrome (XFS) patients.

**Materials and methods:**

Twenty-four patients with unilateral XFS, 20 patients with bilateral XFS, and 23 healthy subjects were enrolled in this study. Eyes with XFS were compared with both fellow eyes and age-matched control subject eyes in terms of mean and segmental RNFL thickness and minimum, mean, and segmental GCC thickness.

**Results:**

In the bilateral XFS group, minimum GCC of the right eye (75.80 ± 11.6 µm) was significantly thinner compared with the right eyes of the control group (81.83 ± 6.6 µm) (P < 0.05). Also, superior RNFL was thinner in the right eye (106.90 ± 16.7 µm) compared with left eye (114.15 ± 18.1 µm) in the bilateral XFS group (P < 0.05). No significant differences in the unilateral XFS group were detected in GCC and RNFL analysis.

**Conclusion:**

Minimum GCC value may be the first parameter affected in the conversion of XFS to exfoliative glaucoma followed by RNFL changes.

## 1. Introduction 

Exfoliation syndrome (XFS), which is characterized by the accumulation of a distinctive fibrillar extracellular material in the anterior segment of the eye, is the most common cause of secondary open-angle glaucoma, especially in the elderly population. XFS can be seen either monocular or binocular and also tends to be asymmetrical (1). Often the disorder is clinically visible unilaterally but it is known that the fellow eye develops the syndrome over the course of time (2). XFS has been shown to cause glaucomatous damage not only by raising intraocular pressure (IOP) but also by diminishing the blood flow in the retina, optic nerve head, and choroidal and retrobulbar vessels (3–6). In this context, patients with XFS are at risk of developing glaucoma (7).

Glaucoma is a progressive optic neuropathy in which neuronal tissue shows gradual degeneration and approximately 30% of the retinal ganglion cells (RGCs) need to be lost in order to give rise to early glaucomatous visual field defects. It is thus important to identify structural neuronal damage as early as possible (8). It is well known that a reduction in retinal nerve fiber layer (RNFL) thickness is an early sign of glaucoma (9–11); however, ganglion cell complex (GCC) thickness, which consists of the combined RNFL, ganglion cell layer (GCL), and inner plexiform layer (IPL), shows similar or even better diagnostic accuracy in detection of early glaucomatous damage (12–15). 

At this point, optical coherence tomography (OCT) seems to be a convenient technique for early diagnosis as some studies suggested that OCT could be superior to other methods such as visual field analyses (16). OCT is a noninvasive imaging technique that provides accurate data on the retinal layers, macula, and optic nerve head (17). Compared with time-domain OCT, spectral-domain OCT (SD-OCT) provides better resolution and reproducibility for image acquisition; therefore, we are now able to obtain high-resolution cross-sectional imaging of the layered structure of the retina and the optic disc. There are some studies about RNFL and GCC analysis using SD-OCT in glaucoma patients or glaucoma suspects and these studies demonstrated high diagnostic accuracy for both (14,18,19).

This study is designed to evaluate RNFL and GCC thicknesses using SD-OCT in both unilateral and bilateral XFS patients and compare the results with fellow eyes and control subjects.

## 2. Materials and methods

### 2.1. Patients and examination technique

Twenty-four patients with unilateral XFS, 20 patients with bilateral XFS, and 23 healthy subjects were enrolled in this retrospective cross-sectional study. 

All participants underwent a comprehensive ophthalmic examination, including full medical history, best-corrected visual acuity (BCVA), slit-lamp biomicroscopy, IOP measurement with Goldmann applanation tonometry, gonioscopy, dilated fundoscopic examination using a 90-D lens, visual field examination with Humphrey field analyzer (HFA; model 750i; Humphrey-Zeiss Systems, Dublin, CA, USA) 30-2 test, and evaluation of RNFL and GCC thicknesses. 

The HFA test was performed two times for all participants in order to minimize the learning effect and results of the second test were used. The test results were considered reliable as long as the fixation losses were <20%, and false positive and false negative errors were <15%. Patients with normal visual field, which was defined as a mean deviation and pattern standard deviation within 95% confidence intervals and glaucoma hemifield test result within normal limits, were included in this study. If any cluster of 3 or more adjacent points depressed more than 5 dB or 2 adjacent points depressed more than 10 dB were detected on HFA test, those with these results were excluded from the study.

### 2.2. Inclusion criteria

To be included in the XFS group, all patients should have exfoliation material at the pupillary border or on the lens capsule either unilaterally or bilaterally without any glaucomatous defects in HFA test. Also, they had to have a mean IOP of <21 mmHg after three measurements over the course of a day using a Goldmann tonometer. BCVA of at least 20/40, spherical refraction less than ±5.0 D, cylinder correction of <2.0 D, and an open angle with the gonioscopy were other inclusion criteria. Patients with any other coexisting ocular diseases such as cataracts that could compromise the quality of the OCT image, uveitis, nonglaucomatous optic disc neuropathies, and retinal diseases were excluded from the study. Patients who had a history of previous intraocular surgery, laser procedures, or previous eye disease were also excluded.

The characteristics of the control group were as follows: IOP <21 mmHg, BCVA of at least 20/40 with refractive error less than ±5.0 D sphere and/or <2.0 D cylinder, no first-degree relatives with glaucoma, no history or evidence of intraocular surgery, normal visual field tests, and a normal optic disc appearance (vertical cup-to-disk ratio 0.6 or less with intereye difference not higher than 0.2, absence of localized or diffuse neuroretinal rim thinning or notching).

### 2.3. Optical coherence tomography analysis

The Cirrus HD-OCT (Carl Zeiss Meditec Inc., Dublin, CA, USA) was used to obtain measurements of GCC and RNFL thickness. An internal fixation target of the OCT device was used while scanning. Each eye was dilated with 2.5% phenylephrine hydrochloride and 0.5% tropicamide before the scanning process. Only good quality images, defined by signal strength of 6 or greater, were used in the analysis.

The circumpapillary RNFL scan protocol was based on 3 consecutive 360° circular scans with a diameter of 3.4 mm centered on the optic disc (Optic Disc 200 × 200 axial protocol). The mean 360° RNFL thickness was defined as the average RNFL thickness in the analysis report. Average RNFL thicknesses in 4 quadrants and over 12 clock hours were also present. The mean quadrant RNFL thickness between 315° and 45° was defined as the temporal, between 45° and 135° as superior, between 135° and 225° as nasal, and between 225° and 315° as inferior. The measurements were aligned on the basis of the right eye orientation. The superior clock hour was 12 o’clock, and the others were designated accordingly clockwise in the right eye and anticlockwise in the left eye. 

The Cirrus HD-OCT Ganglion Cell Analysis (GCA) protocol (Carl Zeiss Meditec, Inc.) automatically segments the ganglion cell and inner plexiform layers (GC-IPL), the two of which go under the name of GCC together, from the remaining retinal layers. Thereafter, the system measures the thickness of these two retinal layers within an elliptic annulus area (vertical radius of 2 mm, horizontal radius of 2.4 mm) centered on the fovea (GCA, Macular Cube 512 × 128 protocol). The GCA protocol gives the minimum GCC thickness, average GCC thickness, and GCC thicknesses in 6 quadrants of the scanned area. Those six quadrants were defined as superotemporal (0–60°), superior (60–120°), superonasal (120-180°), inferonasal (180–240°), inferior (240–300°), and inferotemporal (300–0°). 

### 2.4. Statistical analysis

SPSS 15.0 (SPSS Inc., Chicago, IL, USA) was used for statistical analysis. Categorical data were analyzed by the chi-square test. Either the Wilcoxon signed-rank test or the paired t-test was used to compare the differences between fellow eyes. The Tukey post hoc test was used for multiple comparison. Analysis of covariance (ANCOVA) using age, IOP, and refractive error as covariates was performed to compare the two groups (XFS and control group) for GCC and RNFL thicknesses. P < 0.05 was considered statistically significant.

## 3. Results

Demographic characteristics of the study groups are presented in Table 1. Among the three groups, there were no significant differences in terms of sex, mean IOP, vertical cup/disc ratio, and pattern standard deviation (PSD). The mean age of the control group was shown to be lower compared to both unilateral and bilateral XFS groups (P < 0.05).

**Table 1:** Demographic characteristics of the study groups.

Demographic variable	Bilateral XFS (n = 20)		Unilateral XFS (n = 24)		Control (n = 23)	
			
Sex, number of subjects						
Male	10		12		13	
Female	10		12		10	
Age, years	68.8 ± 7.3		67.8 ± 7.9		58.4 ± 10.6*	
	RE	LE	XFS eye	Non-XFS eye	RE	LE
IOP (mmHg)	16.2 ± 2.4	15.6 ± 2.5	15.9 ± 3.1	14.7 ± 2.9	15.1 ± 2.6	16.3 ± 2.3
Vertical cup/disc ratio	0.33 ± 0.24	0.29 ± 0.21	0.31 ± 0.25	0.34 ± 0.20	0.28 ± 0.29	0.30 ± 0.21
Pattern standard deviation (dB)	1.74 ± 0.58	1.83 ± 0.62	1.79 ± 0.55	1.76 ± 0.44	1.82 ± 0.51	1.79 ± 0.61

Data are expressed as means ± SDs. RE: right eye; LE: left eye. *Statistical significant difference were detected between mean age of XFS groups and control group (P < 0.05).

Table 2 demonstrates the GCC thickness values of superior, superotemporal, infratemporal, inferior, inferonasal, and superonasal quadrants in addition to the mean and minimum values. Minimum GCC thickness of the right eye was significantly lower compared to both the right eye and randomized-selected eye of the control group (P = 0.021 and P = 0.020, respectively). There were no significant differences in the other comparisons.

**Table 2:** GCC thickness values among all groups (μm).

	BilateralXFS RE	BilateralXFS LE	UnilateralXFS eye	Unilateralnon-XFS eye	Control RE	Control LE
GCC thickness (µm)	Mean ± standard deviation					
Minimum	75.80 ± 11.6*	75.75 ± 9.3	71.57 ± 16.5	68.35 ± 16.8	81.83 ± 6.6*	81.70 ± 6.2
Mean	79.80 ± 8.7	79.90 ± 7.8	77.43 ± 12.7	76.39 ± 11.1	83.83 ± 6.3	83.78 ± 6.2
Superior	79.15 ± 10.5	81.10 ± 8.1	78.91 ± 11	76.61 ± 16	84.70 ± 6.6	84.74 ± 6.2
Superotemporal	78.80 ± 8.5	78.55 ± 6.4	78.13 ± 12.5	78.52 ± 9.8	82.91 ± 6.5	83.04 ± 6.1
Inferotemporal	81.15 ± 11.5	78.95 ± 9.6	79.35 ± 13.6	79.74 ± 9.9	83.22 ± 7.1	84.13 ± 6
Inferior	78.45 ± 10.8	78.65 ± 9.5	77.26 ± 13.7	74.09 ± 13.4	82.57 ± 6.9	82.52 ± 7.2
Inferonasal	79.35 ± 9.2	79.95 ± 9.3	76.43 ± 13.7	74.39 ± 17	84.17 ± 7.1	83.39 ± 7.2
Superonasal	81.35 ± 7.8	81.40 ± 8.9	76.39 ± 15.4	76.52 ± 18.9	85.87 ± 6.2	85.22 ± 7.1

*Statistical significant difference was detected between minimum GCC thickness of right eyes of bilateral XFS group and control group (P = 0.021).

Table 3 summarizes RNFL thickness values in 4 quadrants in addition to the mean values. In the comparison of the fellow eyes of the bilateral XFS group, superior quadrant RNFL thickness was significantly lower in the right eyes (106.90 ± 16.7 µm) than in the left eyes (114.15 ± 18.1 µm) (P = 0.036). However, mean nasal quadrant RNFL thickness was significantly higher in the right eyes (74.70 ± 12.1 µm) than in the left eyes (68.25 ± 13.1 µm) of the bilateral XFS group (P = 0.043). No statistically significant difference was detected between the comparison of XFS groups with the control group. Error bar graphs illustrating the mean and 95% confidence interval for each parameter that was significantly different are shown in Figures A–C.

**Table 3:** RNFL thickness values among all groups (μm).

	BilateralXFS RE	BilateralXFS LE	UnilateralXFS eye	Unilateralnon-XFS eye	Control RE	Control LE
RNFL thickness (µm)	Mean ± standard deviation					
Mean	89.60 ± 10.3	90.95 ± 10.9	90.30 ± 9.1	84.35 ± 16.2	91.17 ± 10.5	90.17 ± 8.5
Superior	106.90 ± 16.7*	114.15 ± 18.1*	113.45 ± 12.6	105.45 ± 21.2	119.52 ± 17.1	114.83 ± 28
Inferior	119.5 ± 14.6	118.25 ± 19.2	116.95 ± 17.5	111.23 ± 24.5	118.13 ± 13.8	115.96 ± 13.9
Nasal	74.70 ± 12.1*	68.25 ± 13.1*	69.14 ± 8.7	66.09 ± 9.1	66.57 ± 12.9	63.26 ± 11.3
Temporal	57.70 ± 11.1	63.10 ± 12.7	60.86 ± 10.6	60.95 ± 10.9	62.70 ± 9.2	63.61 ± 10

*Statistically significant differences were detected between the comparison of the right and left eyes of bilateral XFS group (P = 0.036, P = 0.043, respectively).

**Figure 1:**
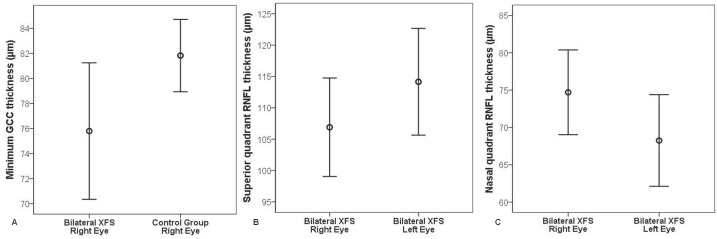
Error bar graphs of the statistically significant values showing the mean value and 95% confidence interval. A) Comparison of the minimum GCC thickness between the right eyes of bilateral exfoliation syndrome (XFS) group and control group (P = 0.021). B) Comparison of the superior quadrant RNFL thickness between right and left eyes of bilateral XFS group (P = 0.036). C) Comparison of the nasal quadrant RNFL thickness between right and left eyes of bilateral XFS group (P = 0.043).

## 4. Discussion

This study demonstrated that right eyes of bilateral XFS patients had significantly thinner minimum GCC values. Due to the asymmetrical course of the disease, even in the bilateral XFS cases, one eye may show earlier signs of progression to exfoliative glaucoma (XFG). Similarly, those right eyes of bilateral XFS patients had lower RNLF thickness in the superior quadrant compared with fellow eyes. These data may be interpreted as a sign of early loss of RNFL reserves due to asymmetrical involvement. The minimum GCC value may be the first parameter affected in the early stages of progression followed by RNFL changes. 

In XFG, IOP can demonstrate a fluctuating pattern in the course of the day, so it is possible to come across normal IOP values during a routine examination. This situation may mislead practitioners while trying to distinguish between XFS and XFG. Hence, it is well known that XFS can show progression to XFG with some unclarified mechanisms and it is crucial to make an early diagnosis of the structural changes to prevent permanent neuronal damage. In this context, RNFL and GCC thicknesses are thought to be good indicators for detecting the progression before glaucomatous visual field defects occur as a result of RNFL thinning and loss of RGCs (20,21).

Several studies have shown that RNFL has diagnostic abilities to differentiate between healthy and glaucomatous subjects (15,18,22). In a study by Ajtony et al., in which eyes with preperimetric glaucoma and primary-open angle glaucoma were involved, a reduction in RNFL layer thickness was shown as an early sign of glaucoma (20). Similarly, Lisboa et al. compared RNFL thickness assessment using SD-OCT with confocal scanning laser ophthalmoscopy (CSLO) by evaluating 134 eyes of 88 glaucoma suspects based on the appearance of the optic disc (23). As a result, they found that RNFL thickness evaluation had a better performance than CSLO in terms of detecting preperimetric glaucomatous damage.

GCC assessment has become a novel trend both in discriminating early glaucomatous changes and glaucoma screening as SD-OCT technologies improve both in terms of hardware and software upgrades. For example, Asrani et al. detected a significant decrease in RGC density before any visual field defects were spotted among a group of glaucoma suspects and glaucoma patients (24). Takagi et al. focused on GCC evaluation with two consecutive studies. In 2011, they drew attention to changes in the thickness of the macular ganglion cell complex in glaucoma patients as emphasizing the need for careful monitorization (25). Macular GCC thickness was designated as a structural parameter for detecting preperimetric glaucoma (15). Similar results were also obtained by some other various studies discussing GCC thickness (9,14,19). 

Naturally, GCC and RNFL thicknesses were also compared with each other concerning their glaucoma discrimination ability. Moreno et al. found the GCC thickness to have a slightly better accuracy to discriminate between early glaucomatous and healthy eyes (26), while Kotowski et al. stated that GCC and RNFL had similar diagnostic ability for perimetric glaucoma but showed no significant differences when comparing healthy eyes with those with preperimetric glaucoma in terms of diagnostic power (27).

Na et al. reported that the ganglion cell-inner plexiform layer (GCIPL), which was affected earlier than RNFL thickness, might serve as an early indicator of glaucomatous structural damage in preperimetric glaucoma (14). However, some studies reported higher sensitivities of RNFL than GCIPL analysis in early glaucoma detection. A study including 56 preperimetric glaucoma cases found that the highest sensitivities at ≥95% specificity were achieved by global pRNFL (51.8%), inner temporal mGCL (37.5%), outer inferior mGCC (28.6%), outer inferior mRNFL (28.6%), and inner temporal mIPL (26.8%) (28). In a population-based study, the diagnostic sensitivity of the minimum GCIPL was reported as 60.5%, which was significantly lower than the sensitivity of the inferior quadrant RNFL (81.8%, P = 0.007) (29). Hammel et al. reported 1.7% decrease in RNFL thickness per year compared to 1.3% decrease in GCIPL per year. They indicated that RNFL may be a more sensitive parameter to detect progression (30). However, in advanced glaucoma cases, there was a significant decrease in GCIPL thickness where no further change in RNFL could occur. These findings support that the relative value of RNFL and GCIPL measurements may vary at different stages of disease, suggesting that GCIPL thickness may be a better index for detecting progression in advanced glaucoma cases (31).

Because it is so important to diagnose the early stages of glaucomatous damage formation in XFS patients, Yüksel et al. discussed the diagnostic ability of RNFL thickness evaluation in patients with unilateral XFS by using OCT (32). Eyes with exfoliation were compared with both fellow eyes and control eyes. They examined the RNFL thicknesses in 4 quadrants and 12 clock-hour segments among all three groups. The study showed that the RNFL values in patients with XFS were significantly thinner than those of controls in all quadrants except the nasal quadrant. In addition, RNFL loss was evident in the 7, 10, and 11 o’clock segments. In addition, RNFL thicknesses of the 1, 2, and 5 o’clock segments in addition to the inferior quadrant in XFS eyes were significantly lower than in fellow eyes. These results suggested that RNFL assessment could be a good method in terms of early detection of the development of glaucomatous damage in eyes with exfoliation. 

Determination of the cut-off values of the annual mean peripapillary RNFL and ganglion cell complex thickness losses is critical in the separation of the normal annual loss from glaucoma progression. A study including 121 healthy participants reported the annual decrease in mean RNFL thickness as 0.365 µm (33). Regarding quadrant analysis, the greatest decrease in mean RNFL thickness was seen in the lower quadrant (0.575 µm/year), while the least decrease occurred in the nasal quadrant (0.141 µm/year). Another report studying 191 eyes indicated that overall GCC thickness decreased by 0.25 ± 0.05 µm per year while the overall RNFL thickness decreased by 0.14 ± 0.07 µm per year with longitudinal analyses (34). A recent study including a larger number of subjects (295 subjects) revealed that mean macular GCIPL decreased by 0.12 µm with every year of age and 1.61 µm per decade (35). Any decrease higher than these defined cut-off limits may be interpreted as a sign of progression. 

The major limitations of this study were relatively small sample size and retrospective nature. Further studies on patients with exfoliation with greater sample size are required in order to identify the correlation between the development of glaucomatous damage in eyes with exfoliation and alteration of GCC and RNFL thickness. 

In conclusion**, **minimum GCC value may be the first parameter affected in the course of glaucoma progression in XFS followed by RNLF changes. Periodical repetition of GCC and RNFL analysis may be important in the early detection of the initiation of glaucomatous damage.
